# Ultra-stable sub-meV monochromator for hard X-rays

**DOI:** 10.1107/S1600577515012230

**Published:** 2015-07-17

**Authors:** T. S. Toellner, J. Collins, K. Goetze, M. Y. Hu, C. Preissner, E. Trakhtenberg, L. Yan

**Affiliations:** a Advanced Photon Source, Argonne National Laboratory, Argonne, IL 60439, USA

**Keywords:** high energy-resolution, monochromator, europium, nuclear resonance, cryostat

## Abstract

A high-resolution silicon monochromator suitable for 21.541 keV synchrotron radiation is presented that produces a bandwidth of 0.27 meV. The operating energy corresponds to a nuclear transition in ^151^Eu. The first-of-its-kind, fully cryogenic design achieves an energy-alignment stability of 0.017 meV r.m.s. per day, or a 100-fold improvement over other meV-monochromators, and can tolerate higher X-ray power loads than room-temperature designs of comparable resolution. This offers the potential for significantly more accurate measurements of lattice excitation energies using nuclear resonant vibrational spectroscopy if combined with accurate energy calibration using, for example, high-speed Doppler shifting. The design of the monochromator along with its performance and impact on transmitted beam properties are presented.

## Abstract 2

A 0.27 meV-bandwidth monochromator for 21.5 keV synchrotron radiation demonstrates exceptional stability using cryogenic stabilization and active feedback control.

## 1. Introduction   

Synchrotron-based high-resolution X-ray spectroscopy is used in many research fields to characterize and study the behavior of systems at the atomic level. There are three unique high-resolution X-ray spectroscopies in the hard X-ray region (5–30 keV). One is inelastic X-ray scattering (IXS), which is used to measure lattice dynamical excitations and how they disperse by detecting very small energy and momentum transfers when X-rays scatter off a crystal (Burkel, 2000 ▸). The other two spectroscopies involve the excitation of nuclear resonances in various isotopes (*e.g.* ^57^Fe, ^119^Sn, ^151^Eu, *etc.*) that exist in this spectral region. One technique is incoherent inelastic nuclear resonant scattering also known as nuclear resonant vibrational spectroscopy (NRVS) and is also used to probe lattice excitations, but is sensitive to their local influence at the resonant isotope only (Chumakov & Sturhahn, 1999 ▸). The other is synchrotron Mössbauer spectroscopy (SMS), which uses the Mössbauer effect, and probes the hyperfine interactions present at the resonant isotope *via* coherent elastic scattering of synchrotron radiation (Gerdau & de Waard, 2000 ▸). All of these techniques require monochromatization of synchrotron radiation to millielectronvolt or sub-millielectronvolt bandwidths.

High-resolution monochromatization of hard X-rays has witnessed significant advances over the past few decades motivated by these X-ray techniques and the potential for higher data rates created by the advent of third-generation synchrotron sources. In the 5–30 keV spectral region, numerous configurations of multiple crystals, typically silicon, have been developed to produce high energy resolution (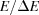 > 10^6^) with usable spectral efficiency (Faigel *et al.*, 1987 ▸; Ishikawa *et al.*, 1992 ▸; Toellner, 1996 ▸; Chumakov *et al.*, 1996 ▸; Yabashi *et al.*, 2001 ▸; Toellner *et al.*, 2011 ▸). Improving energy resolution to 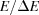 ≃ 10^8^ and beyond in a manner that is practical for performing spectroscopy requires significant improvements beyond just crystal configuration. Stability, calibration and efficiency warrant careful attention and pose a significant challenge at higher resolutions.

Measured energy resolution has contributions from both energy bandwidth produced by the optics and energy-alignment instabilities. To achieve higher resolutions to well below sub-meV bandwidths, it becomes increasingly important to improve both. For meV-bandwidth high-resolution monochromators (HRMs), the instabilities are typically small enough compared with the bandwidth for adequate periods of time that their impact can be managed. As bandwidths are reduced, however, the instabilities can become a significant contribution to the effective energy resolution.

Improving bandwidth and stability along with efficiency can be achieved with a combination of crystal configuration and cryogenic stabilization, which involves operating the diffracting crystals at a temperature where the coefficient of thermal expansion vanishes. This stabilizes the lattice parameter and thus the diffracted wavelength against small to moderate thermal influences, and, because of the lower temperatures, also leads to increased Debye–Waller factors that improve reflectivity profiles and thus optic efficiency. The reduced thermal expansion coefficient along with the greater thermal conductivity of single crystals at cryogenic temperatures also contributes to a greater tolerance to X-ray power loading from stronger sources. For X-ray energies above 20 keV, cryogenic stabilization has demonstrated improved efficiency of meV-bandwidth X-ray optics (Toellner *et al.*, 2006 ▸, 2011 ▸), but stability proved to be only somewhat better owing to designs that lacked fully integrated control of thermal and mechanical influences.

We present here a solution based upon a fully cryogenic design that presents a nearly 100-fold improvement in energy stability over room-temperature designs (which are typically a few meV per day) while still obtaining very high energy resolution. It does this by integrating cryogenic stabilization with active feedback control on both temperature and crystal angles. The monochromator produces the highest resolution using crystal optics ever achieved to date for nuclear resonant scattering measurements at the 21.541 keV level in ^151^Eu (Leupold *et al.*, 1996 ▸; Shvyd’ko *et al.*, 2002 ▸; Barla *et al.*, 2004 ▸; Toellner *et al.*, 2006 ▸; Yoda *et al.*, 2007 ▸; Sergueev *et al.*, 2011 ▸). The significantly improved design allows more stable energy alignment at a fixed energy; for example, to operate at a nuclear transition energy for an indefinite period of time as is required for SMS measurements. It also allows more reliable energy scanning, which for NRVS is critical for accurate measurement of vibrational energies. Thus, the conceptual design of the HRM can be duplicated at other energies; for example, at energies corresponding to X-ray analyzers for IXS, to improve the measurement of vibrational spectra. Future synchrotron sources may produce higher spectral flux and this would create the opportunity for higher data rates as well as make higher resolution practical, but it would also place a higher X-ray power load on the crystal optics. The use of cryogenic stabilization with active feedback control is an approach that can tolerate higher X-ray loads while still producing very high energy resolution with excellent alignment stability.

After presenting the design and the measured performance, we discuss the results along with additional effects on the transmitted synchrotron radiation beam that originate from multiple asymmetrically cut crystal configurations and have important consequences for applications.

## 2. Design   

The design involves a four-reflection silicon-crystal design that is cooled to the temperature at which the coefficient of thermal expansion for silicon vanishes (123 K). It utilizes a specially designed cryogenic platform that houses the HRM and positions it into the X-ray beam. The monochromator itself incorporates active feedback control of crystal angles to achieve unprecedented energy alignment stability. We first present the design of the cryo-platform, and then present that of the HRM itself.

### 2.1. Cryo-platform   

A specially designed cryostat with a copper chamber (40 cm × 20 cm × 20 cm) houses the entire HRM including mechanics, position and temperature sensors, piezo actuators, ion-chambers and a PIN photodiode. The chamber is installed inside a vacuum vessel for thermal isolation. Cold helium gas flows through channels in five of the six walls of the chamber to effect the cooling. Helium gas is cooled *via* a two-stage heat exchanger. The primary stage consists of copper coils immersed in a liquid-nitrogen bath at atmospheric pressure. The secondary stage uses the cold gas returning from the monochromator chamber to pre-cool the supply of room-temperature helium gas before it enters the primary heat exchanger. This improves the overall efficiency of the heat exchanger.

A pump at room temperature recirculates the helium gas creating a closed cycle. Pulsation dampeners on both the inlet and the outlet sides of the pump mitigate a major source of vibrations that would otherwise significantly deteriorate the performance of the HRM.

The temperature of the cryostat is actively controlled using a mass-flow controller that varies the He-gas flow rate to maintain a target temperature as measured by platinum resistance thermometers (Pt-111) that are placed in the gas stream. The mass-flow controller operates at room temperature and is located between the pump and the heat exchanger. After a cool-down phase that takes approximately 24 h, the mass-flow controller maintains the cryostat temperature within 2 mK of its target temperature with a helium-gas flow-rate of approximately 77 L min^−1^. The liquid-nitrogen bath loses approximately 39 L day^−1^ to evaporation.

**Figure 1:**
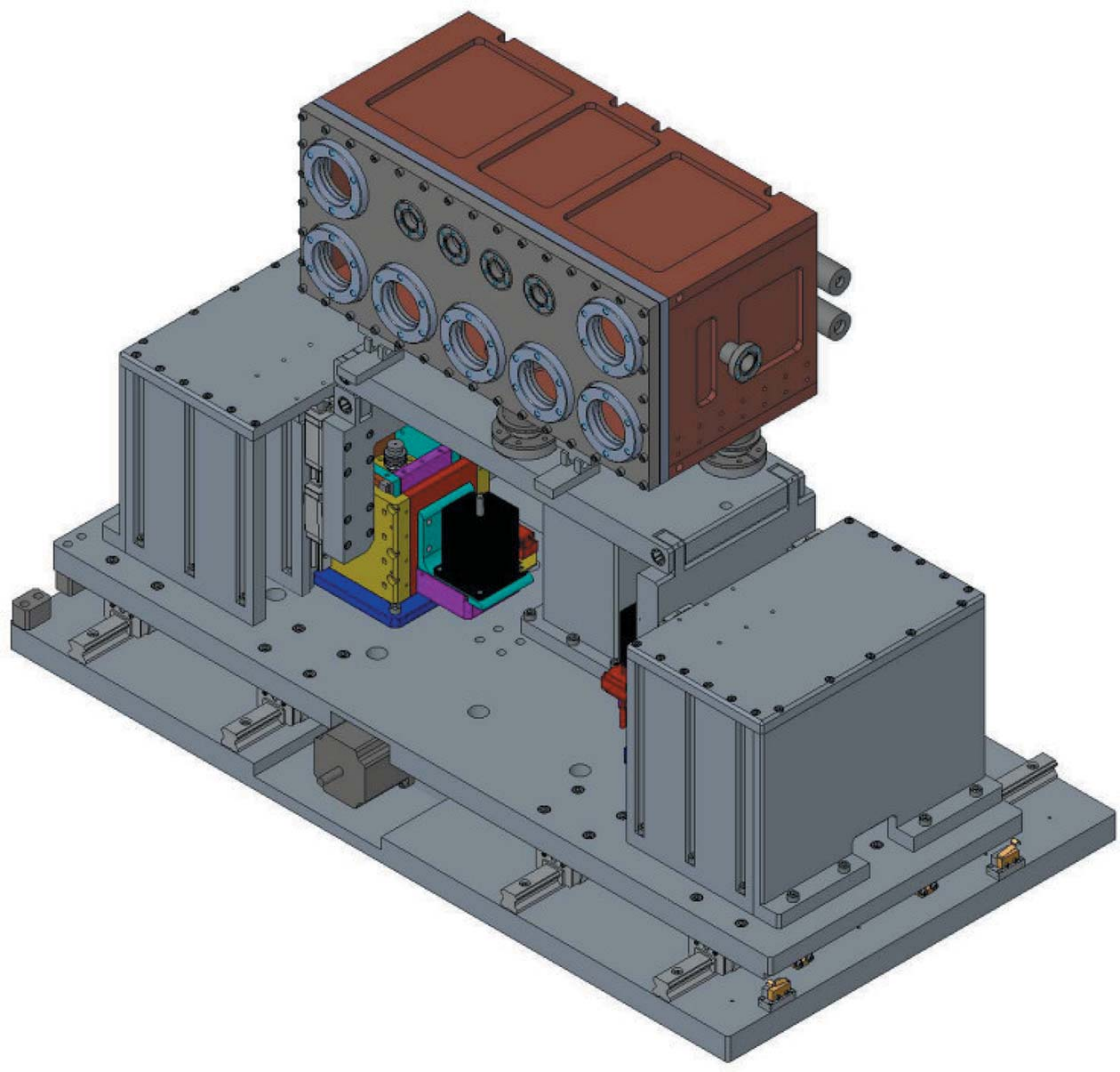
Drawing of the cryo-platform including the gas-cooled chamber (top) that houses the HRM, and positioners for aligning the HRM to the X-ray beam (bottom). The outer vacuum vessel is not shown.

The HRM is placed inside the copper chamber on its own kinematic mounting platform that is partially decoupled from the chamber *via* highly flexible bellows around each of three mounting spheres. The mounting platform allows the height and overall pitch of the HRM to be controlled from a driving structure that is outside the chamber, while also diminishing the impact of chamber vibrations on the alignment and energy stability of the HRM. The copper chamber contains nitrogen gas at 150 mbar (absolute) to facilitate thermal transport between the HRM and the chamber walls. The gas also acts as an ionizing medium for three ion chambers that allow for *in situ* flux monitoring of the diffracted beam from the first three crystals. A drawing of the cryo-platform is shown in Fig. 1 ▸.

### 2.2. Monochromator   

**Figure 2:**
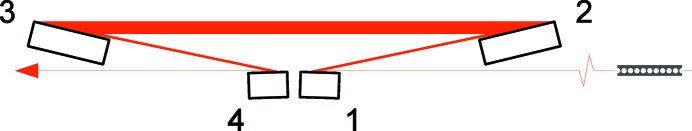
Schematic of the crystal arrangement for the HRM including a beryllium compound refractive lens that is placed 30 m upstream. Numbers refer to crystal reflections and are referenced in the text.

**Figure 3:**
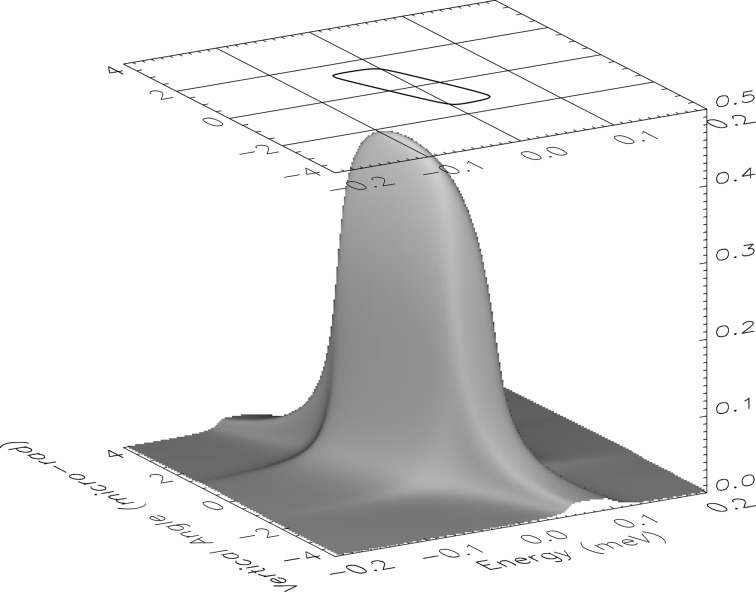
Calculated bare-optic transmission function of HRM for 21.541 keV σ-polarized photons. The peak transmission is 43%, and the contour is drawn at 50% of this value.

The HRM employs four silicon crystals aligned to the (12 12 8) lattice reflection in a (+, −, −, +) arrangement as shown in Fig. 2 ▸. This crystal configuration was chosen because it allows a compact inline design that can achieve very high energy resolution. At 123 K, the Bragg angle for 21.541 keV X-rays (λ = 57.6 pm) is 83.928°. All crystals have the surface normal of the diffracting surface within the diffraction plane and at angles 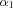 = 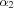 = 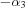 = 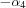 = −82.16° relative to the [12 12 8] lattice vector, leading to asymmetry parameters for each crystal of 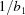 = 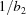 = 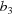 = 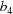 = 7.8. Note that 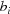 = 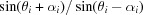 and that 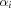 > 0 implies that the incident angle is greater than the Bragg angle. This arrangement has been employed previously using different crystal parameters to produce a monochromator at room temperature for 14.4 keV X-rays (Yabashi *et al.*, 2001 ▸). The individual crystals were cut from a boule of hyperpure monocrystalline silicon with a resistivity in excess of 70 kΩ cm. The crystals were fabricated such that the diffraction plane coincides with a crystallographic zone (zone axis 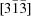) that reduces the influence from other lattice reflections at this X-ray energy. The monochromator transmission function *versus* vertical angle and X-ray energy of incident beam is shown in Fig. 3 ▸.

**Figure 4:**
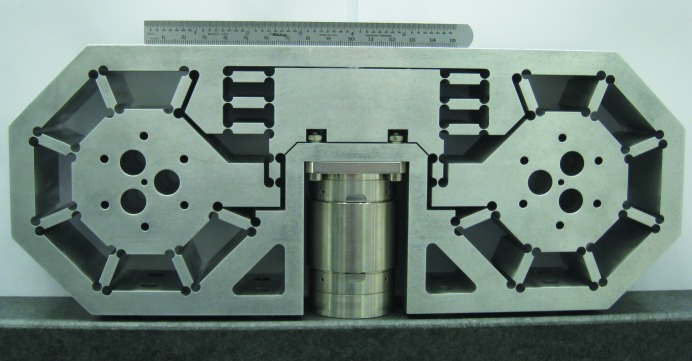
Photograph of the monolithic stage using flexures for energy positioning. Both axes are counter-rotated to effect a controlled rotation of all four crystals with a single actuation. The centrally located cylindrical component is the coarse actuator (PI N-215) with its vertical drive shaft hidden from view.

The mechanical structure for supporting the crystals and controlling their angles is based on piezoelectric-driven weak-link flexures fabricated from grade 7075 aluminium with capacitive sensors for active feedback control. Crystals #1 and #2 are mounted on a single monolithic flexural plate with a pair of capacitive sensors to monitor their pitch (Bragg) angle relative to each other. Piezoelectric actuation on the pitch of crystal #2 allows the first two crystals to be aligned relative to each other, while the pair of capacitive sensors are used as part of an active feedback system that controls and maintains any target angle. This forms a two-crystal subassembly. Similarly, crystals #3 and #4 are mounted on a separate monolithic flexural plate and form a second two-crystal subassembly with active feedback control that adjusts the pitch of crystal #4 to maintain its alignment relative to crystal #3. These two flexure subassemblies are mounted on a third, more complicated, monolithic flexural plate shown in Fig. 4 ▸. The third flexural plate is the energy-scanning stage and allows the pair of crystal subassemblies to be counter-rotated relative to each other in such a way that allows wavelength changes without altering the overall angular alignment of the HRM to the beam. A third piezoelectric transducer with active feedback using a third pair of capacitive sensors rotates both of these crystal pairs relative to each other to effect energy scanning over a range 0.35 eV. This allows a single backlash-free actuator to control the energy alignment of the HRM. A coarse piezo-based actuator (Physik Instrumente model N-215) is in series with the third piezoelectric transducer that allows motion over a 7 eV energy range. The coarse actuator can be seen in Fig. 4 ▸. Other motions that align the HRM to the X-ray beam, such as vertical and horizontal translations along with an overall pitch alignment, are driven *via* a combination of piezoelectric actuators and stepper motors located outside the cold environment.

**Figure 5:**
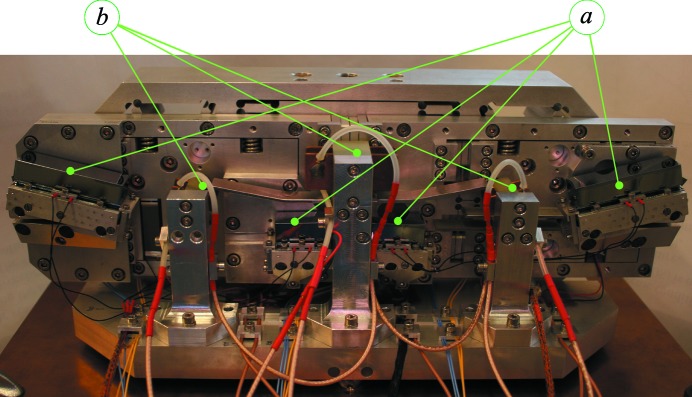
Photograph of the HRM. Silicon crystals (*a*), and ionization chambers (*b*) are marked. The largest dimension is approximately 38 cm.

Flux monitoring is integrated into the design to allow the crystals to be aligned initially to their Bragg reflections during cryogenic operation, while the capacitive sensors are used for active feedback control to maintain the crystal angles. A PIN photodiode positioned downstream of crystal #1, but inline with the incident beam, is used for monitoring the vertical position of the HRM relative to the X-ray beam. Platinum resistance thermometers (Pt-111) are placed in close proximity to each crystal for monitoring temperatures. The complete monochromator assembly with integrated flux monitors is shown in Fig. 5 ▸.

The HRM has an angular acceptance of 2.9 µrad and a vertical spatial acceptance of 0.2 mm. Acceptances in the horizontal are approximately 100 times larger. To compensate for the small vertical acceptances, a beryllium compound refractive lens (CRL) consisting of 12 one-dimensional circular (radius = 0.53 mm) cylindrical lenses (focal length = 30 m) was positioned in the beam 34 m from the source, while the HRM was positioned 64 m from the source (Baron *et al.*, 1999 ▸; Chumakov *et al.*, 2000 ▸). This reduced the vertical size and divergence of the beam, and enhanced the transmission through the HRM by approximately 50%.

## 3. Testing   

We measured the performance of the HRM at the 3-ID undulator beamline of the Advanced Photon Source. Characterization of the HRM involves a measurement of its energy-resolution function, its efficiency, and the stability of its energy alignment.

It is necessary to calibrate the single-axis energy-positioning system to reflect accurately the correct changes in energy alignment. This is important because NRVS data require scanning the energy, which, when taken relative to the nuclear transition energy, represents the vibrational excitation energy (Chumakov & Sturhahn, 1999 ▸). Converting changes in crystal angle to changes in energy alignment requires a calculable angle-to-energy conversion factor of 0.436 µrad meV^−1^. A measurement of the nuclear resonant absorption spectrum of ^151^Eu_2_O_3_ over a 120 meV range allowed calibration of the energy scale after comparison with previous measurements (Toellner *et al.*, 2006 ▸).

**Figure 6:**
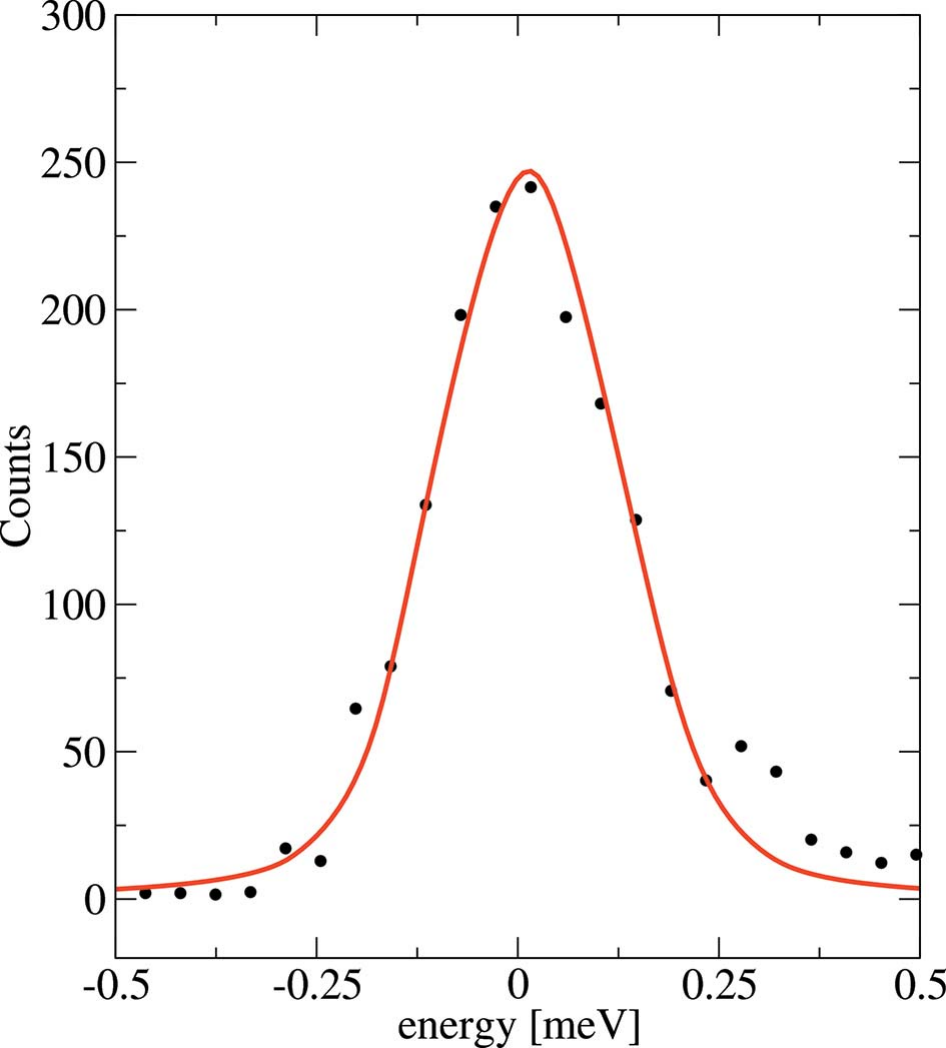
Resolution function of the monochromator measured at 21.541 keV using nuclear resonant scattering from ^151^EuS. The resolution function is measured under normal operating conditions with a cooling-gas flow rate of 1.3 l s^−1^ (solid circles) and has a FWHM of 0.27 meV. The solid line is a shape function based upon two-beam dynamical diffraction.

The spectral distribution of the transmitted X-rays, or resolution function, of the HRM was measured directly using nuclear forward scattering (NFS) from ^151^EuS powder. NFS is a coherent elastic scattering channel with a sub-µeV spectral response, and so functions effectively as a delta function in energy that can be employed for measuring the energy resolution function of the HRM. The measured resolution function is shown in Fig. 6 ▸ and has a full width at half-maximum (FWHM) of 0.27 meV. Contributions to the width of the energy resolution from gas-flow-induced vibrations were tested by temporarily stopping the cryostat’s gas flow during measurements; no broadening was detected. There existed sources of vibration other than flow, and all of these impacted the stability of the three angular alignments relevant for energy alignment of the HRM. The angular fluctuations of the three angular motions were reflected in the capacitive-sensor read-back values and each distribution had a root mean square (RMS) of 5.2 nrad (crystal #2), 4.0 nrad (crystal #4) and 6.4 nrad (energy stage). The impact of each angular instability on energy positioning was 0.007 meV, 0.001 meV and 0.015 meV, respectively. Other potential contributions to energy broadening that are measurable include crystal surface flatness (5 µrad RMS) and temperature fluctuations (2 mK RMS), but these are both small enough to have negligible impact.

The incident flux was 1.8 × 10^13^ photons s^−1^ within a bandwidth of 1.6 eV and a beam size of approximately 0.65 mm (V) × 2.0 mm (H) FWHM, but this was reduced to 6 × 10^12^ photons s^−1^ with a 0.2 mm vertical aperture to correspond to the spatial acceptance of the HRM. With this aperture, but without the CRL, the transmitted flux was 3.5 × 10^7^ photons s^−1^. After insertion of the CRL upstream, the transmitted flux increased to 5 × 10^7^ photons s^−1^. The measured spectral efficiency, if defined as the ratio of spectral flux after the CRL-HRM to that before without apertures, was 1.6%.

**Figure 7:**
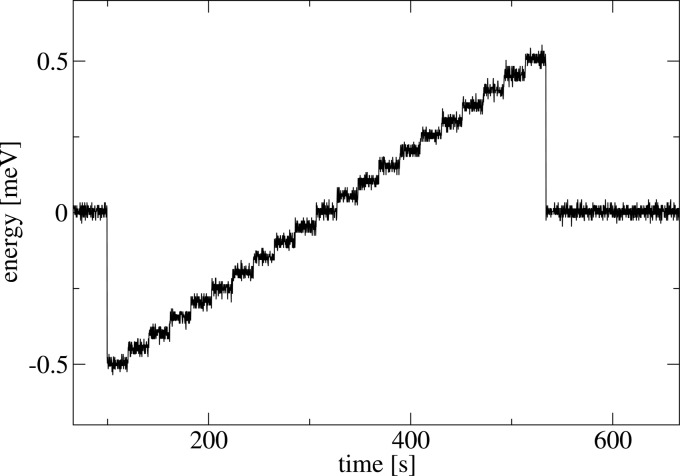
Energy read-back values during an energy-stepped scan of 50 µeV intervals. Energy positions are reached in approximately 50 ms.

The HRM exhibited excellent energy-alignment, or wavelength stability, and this was evident upon aligning the HRM to the nuclear resonance in ^151^Eu. Despite the narrowness of both the nuclear resonance and the resolution function, the HRM maintained its alignment to the nuclear resonance for an indefinitely long time (at least a day). Energy alignment was also unaffected by changes in X-ray power load, *e.g.* by temporarily removing the incident X-ray beam (20 mW). In addition to the long-term stability, Fig. 7 ▸ shows a sequence of 0.05 meV steps and demonstrates the scanning capability of the instrument. From the three angular distributions of capacitive-sensor read-back values, the overall energy-alignment instability has a standard deviation of 0.017 meV RMS. Changes in energy position execute within approximately 50 ms.

## 4. Discussion   

### 4.1. Energy resolution   

From the measured energy resolution (*cf*. Fig. 6 ▸), the bandwidth has a FWHM of 0.27 meV. This represents a resolution (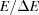) of 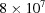, and a longitudinal coherence length (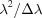) of 4.6 mm. Ideal crystals under ideal conditions would produce a bandwidth of 0.075 meV FWHM based upon two-beam dynamical diffraction simulations. We expected 0.1–0.13 meV FWHM overall including contributions from both measured and non-measured sources. The measured sources include mainly vibrations of 0.040 meV FWHM with sources from surface flatness and temperature instabilities being negligible. The unmeasured sources of energy broadening include strain from fabrication and mounting, as well as from intrinsic lattice imperfections; the latter being expressed as a variation in lattice spacing (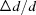) and estimated to be approximately 1–2 × 10^−9^ RMS, or 0.05–0.1 meV FWHM at 21.5 keV, based on previous room-temperature monochromators that have achieved energy resolutions greater than 10^8^ (Toellner *et al.*, 2001 ▸; Yabashi *et al.*, 2001 ▸).

The factor of approximately 2.4 discrepancy between measured and expected bandwidth can have contributions from residual strain during fabrication, intrinsic lattice imperfection or mounting strain. The latter is improbable owing to the lack of any clamping; the crystals were simply resting on polished mounts. Remnant strain cannot be ruled out despite many attempts at etching and polishing the crystal diffracting surfaces. Intrinsic lattice imperfections are expected to be small for hyperpure silicon, but there is a possibility that the degree of lattice imperfection (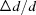) may increase by lowering the temperature of the crystals. Such an effect would have its origin in the isotopic distribution present in natural silicon: 92.23% ^28^Si, 4.67% ^29^Si and 3.10% ^30^Si (Lederer & Shirley, 1978 ▸).

Isotopic defects affect the crystal lattice both dynamically and structurally. The dynamical effects from different isotopes include slight changes in phonon mode frequencies, Debye–Waller factors, mean-square displacements, and the like. These can have a very small impact on, for example, thermal diffuse scattering and X-ray reflectivity. Structural effects have their origin in the distribution of interatomic bond lengths for different isotope combinations. The difference in the average lattice parameter between different mono-isotopic silicon crystals increases as temperature is lowered from room temperature (*e.g.* Pavone & Baroni, 1994 ▸; Herrero, 1999 ▸; Wille *et al.*, 2002 ▸) and is maximal near 75 K (Wille *et al.*, 2002 ▸). Thus, interatomic bond lengths are naturally different for different isotopes of silicon, which is related to their unequal mean-square atomic displacements brought about by both zero-point motion and anharmonicity. An isotopic mixture will thus possess a distribution of bond lengths that ought to widen (narrow) as temperature is lowered (raised) from room temperature. Owing to the fact that structural defects in crystalline silicon are exceptionally small to begin with, *viz.* 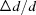 ≃ 10^−9^, it is conceivable that a slight widening in the distribution of interatomic bond lengths due to different isotope combinations may have an observable impact on 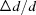. Although it might be somewhat surprising if such an effect would explain our results, the smallness of the required effect (a few p.p.b. is required) makes it difficult to discount. The authors are unaware of any previous suggestion of isotopic distribution introducing a temperature dependence of 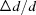 and how that may affect the energy resolution achievable with crystal monochromators operating at lower or higher temperatures.

### 4.2. Spectral efficiency   

Spectral efficiency is determined by a combination of HRM and incident beam properties. The measured spectral efficiency of 1.6% is approximately a factor of four less than theoretically expected. We anticipated to gain a factor of 2.5 in transmitted flux with the introduction of the CRL instead of the 1.5 that we observed, and we attribute this to the combined effect of the CRL and the imperfect diamond double-crystal monochromator of this beamline. We also did not expect the additional energy broadening of approximately 2.4 as described above. These losses taken together (a factor of four loss), if mitigated, would have resulted in a spectral efficiency of 6.5%, which is the theoretically expected result.

It is important to understand all the contributions to the low spectral efficiency in order to consider ways to improve it. The main contributor to the low efficiency is the mismatch between the spatial and angular acceptance of the HRM and the size and divergence of the X-ray beam (factor of 11 loss without CRL, or factor of 7.5 loss with CRL). A secondary contributor is the unexpected (factor of 2.4 loss) and expected (factor of 1.5 loss) broadened bandwidth (factor of 3.6 loss *en toto*). A third contributor is the expected on-axis transmission (43%) that results from combining the reflectivities of four, asymmetrically cut, high-*Q* crystal reflections (factor of 2.3 loss).

Despite the less than expected efficiency, it is still notably greater than a room-temperature design (Toellner *et al.*, 2001 ▸) that achieved a slightly greater energy resolution. In addition to gains introduced by the CRL, the higher efficiency is mainly a consequence of the lower operating temperature, because the Debye–Waller factor increases from 0.24 at room temperature to 0.46 at 123 K, and this results in an increase in angular acceptance by the same ratio as well as an increase in the reflectivity of all four asymmetric reflections from 0.66 to 0.82. In this X-ray region, but especially at higher energies where room-temperature Debye–Waller factors are even lower, the efficiency gains over room-temperature designs by operating at cryogenic temperatures can be substantial. Also, for lower energy-resolution instruments (1–2 meV) that may have less difficulty attaining the designed bandwidth, additional performance gains should be expected.

### 4.3. Stability   

Controlling the wavelength and intensity of the transmitted X-ray beam is critical for operating the HRM owing to the narrowness of the energy bandwidth involved. After the HRM is aligned to the 21.541 keV nuclear transition in ^151^Eu it maintains that energy position indefinitely and without user assistance. Thus, any drifting is, at most, a small fraction of the HRM bandwidth per day, or a few tens of microelectronvolts per day. This amounts to a 100-fold improvement when compared with room-temperature designs that typically experience drifting of approximately a few millielectronvolts per day. The stability should be preserved for X-ray power loads up to 0.1 W, while higher loads would require further study. Changes in the incident beam do not affect the energy alignment of the HRM owing to the fact that the transmitted wavelength is determined by the relative angle between crystals, but the transmitted flux is only as stable as the incident beam is in terms of angle, position and its distributions. During synchrotron operating modes whereby storage ring current is fixed within a few percent, and when upstream beamline components were stable, we observed the transmitted flux to be stable within a few percent as well.

The excellent stability stems from three design concepts. The first involves the use of multiple active feedback control systems whereby a piezoelectric actuator is periodically (100–300 Hz) adjusted to maintain a preset angle position as measured using the difference of two capacitive sensors. The second involves excellent temperature control that maintains the entire monochromator to within a few mK of a preset temperature. This affords excellent mechanical stability in the absence of a changing X-ray load. The third involves choosing the operating temperature to coincide with silicon’s zero-thermal-expansion temperature of 123 K. This requires the additional complexity of cryogenics, but stabilizes the crystal lattice additionally against moderate changes in X-ray load.

Owing to the exceptional stability, the use of backlash-free mechanics and the real-time energy-position sensing, performing energy scans on the fly should be possible. In addition to removing lost time that is inherent in step scans, on-the-fly scans would limit the impact of incident beam fluctuations that occur on a time period that is large compared with the sweep duration. This would result in more accurate measurements that would improve NRVS. Further improvements to NRVS measurements can be obtained by exploiting the energy alignment stability to produce a more reliable energy scale, but this requires relative energy-alignment calibration (relative to the nuclear transition energy) with an absolute uncertainty that is small compared with the HRM bandwidth. This can be achieved with high-speed (∼1 km s^−1^) Doppler shifting of a nuclear resonant material, *e.g.* in the form of a spinning disk made from a high-strength material that contains or supports the nuclear resonant material. By observing elastic nuclear resonant scattering from a fast-rotating material, the energy position of the HRM can be verified with very high accuracy from a measurement of the rotational frequency (ω) and the tangential distance between the beam and the axis of rotation (**r**), using 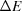 ≃ 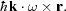 Thus, combining the current highly stable HRM design with a high-speed rotating material could produce a highly reproducible calibrated energy scale that would yield uncertainties in lattice excitation energies in the range 0.01–0.1 meV, and would be independent of material properties and assumptions regarding the mechanical motion. This has the potential to produce much higher quality spectroscopic data using NRVS.

### 4.4. Side effects   

Owing to the use of asymmetric crystal reflections, synchrotron radiation pulses transmitted by the HRM are modified in a number of ways. Longitudinally, the pulse is both stretched and sheared. The longitudinal, or temporal, stretching originates from the bandwidth reduction and is given by 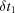 ≃ 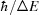. This is approximately 3 ps, and is much smaller than the incident pulse itself (∼80 ps FWHM). Longitudinal pulse shear (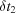) is estimated from the difference in optical path length (OPL) of extremal rays. For flat, undistorted, symmetrically cut crystals, there is no variation in the OPL and therefore no temporal shear. For an asymmetrically cut crystal where the surface normal is not collinear with the lattice vector, different portions of an X-ray beam will experience a different OPL. An X-ray optic composed of multiple crystals may produce some or no net shear depending upon the individual crystal parameters and the arrangement of the crystals relative to each other. For *N* flat crystals with coplanar lattice vectors and surface normals (of the diffracting surfaces), the net temporal shear is given by
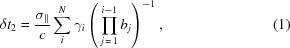
where 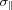 is the lateral separation of rays, or accepted beam size (in the plane of the surface normals), *c* is the speed of light, and 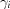 is given by 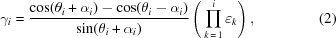
with 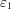 = 1, 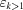 = 1 for crystal reflections in a (+, −) arrangement with respect to the previous reflection, and 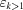 = −1 for crystal reflections in a (+, +) arrangement. Note that the use of equivalent crystal reflections with opposite asymmetry angles and arranged in a (+, −) geometry (*e.g.* a channel-cut) results in no pulse shearing. The HRM produces a very large temporal shear of approximately 740 ps, which is significantly larger than either the temporal stretch or the incident pulse length. In the configuration employed for the HRM (*cf*. Fig. 2 ▸), the top of the transmitted beam lags the bottom by this amount. For coherent elastic NRS that measures the time-differential decay of a nuclear excitation with typically sub-nanosecond time resolution, this aspect of the temporal broadening may be significant.

The use of asymmetric crystals also introduces dispersion into the beam (*i.e.* transverse momentum that is wavelength-dependent) that affects the transmitted beam divergence. Like longitudinal stretching, this is also proportional to the gradient in the optical path length introduced by asymmetrically cut crystals. A general expression for the dispersion contribution to the angular divergence from *N* flat, asymmetrically cut, crystals with all lattice vectors and diffracting surface normals being coplanar is given by
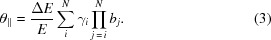
This expression accounts for divergence broadening along the plane of the surface normals. There is no divergence broadening that is transverse to that plane. Because this dispersive effect is proportional to energy bandwidth, there is no significant divergence broadening for the sub-µeV-bandwidth radiation typically encountered in SMS. The combination of two equivalent crystal reflections with opposite asymmetry angles and arranged in a (+, −) geometry is also free of this dispersion. For the current HRM, the transmitted beam divergence has a calculated contribution of 14 µrad owing to the 0.27 meV bandwidth. This is substantially larger than the accepted incident beam divergence (2.9 µrad) and larger than expected because of the additional energy broadening. As a result of altering the beam divergence even though the product of the asymmetry parameters is unity, this dispersion has the effect of producing a vertically broadened virtual source that may adversely affect the performance of downstream focusing optics (Huang *et al.*, 2012 ▸).

Although this angular dispersion is typically undesirable, there exists the possibility to exploit the chromaticity dependence in the dispersion to assist in the filtering of coherent elastic nuclear resonant scattering from a meV-bandwidth synchrotron beam. For a HRM that exhibits this angular dispersion, the full bandwidth of the transmitted beam will have a larger divergence (parallel to the diffraction plane) than the very narrow sub-µeV-bandwidth beam associated with the nuclear resonant radiation.

Consequently, additional filtering of the narrow-bandwidth nuclear resonant radiation can be obtained either with the use of an angular slit in the form of a crystal reflection or, when combined with focusing optics, with a spatial slit positioned at, or near, the focal spot of the resonant radiation (Kohn *et al.*, 2009 ▸).
